# In Vitro Psilocybin Synthesis by Co‐Immobilized Enzymes

**DOI:** 10.1002/chem.202501037

**Published:** 2025-04-21

**Authors:** Tim Schäfer, Alexander Sherwood, Thomas Kirkland, Thomas Krüger, Jakob Worbs, Olaf Kniemeyer, Markus Gressler, Dirk Hoffmeister

**Affiliations:** ^1^ Pharmaceutical Microbiology Friedrich Schiller University Beutenbergstrasse 11a Jena Germany; ^2^ Pharmaceutical Microbiology Leibniz Institute for Natural Product Research and Infection Biology – Hans Knöll Institute Beutenbergstrasse 11a Jena Germany; ^3^ The Usona Institute 2780 Woods Hollow Road Madison WI USA; ^4^ Research & Development Promega Corporation 2800 Woods Hollow Road Madison WI USA; ^5^ Molecular and Applied Microbiology Leibniz Institute for Natural Product Research and Infection Biology – Hans Knöll Institute Beutenbergstrasse 11a Jena Germany

**Keywords:** biotechnology, enzyme, immobilization, natural products, psilocybin

## Abstract

Advanced clinical trials investigate the *Psilocybe* magic mushroom natural product psilocybin as a treatment against major depressive disorder. Currently, synthetic material is used to meet the demand for legitimate pharmaceutical purposes. Here, we report an in vitro approach to biocatalytically produce psilocybin on a solid‐phase matrix charged with five covalently bound biosynthetic enzymes. These enzymes include three *Psilocybe* enzymes: IasA*, an engineered l‐tryptophan decarboxylase/aromatic aldehyde synthase, the 4‐hydroxytryptamine kinase PsiK and the norbaeocystin methyltransferase PsiM, along with *Escherichia coli* nucleosidase MtnN and adenine deaminase Ade. In a proof‐of‐principle experiment, this enzyme‐charged resin allowed for quantitative turnover of 4‐hydroxy‐l‐tryptophan into psilocybin. This facile process i) represents a sustainable approach with reusable enzymes, ii) circumvents the drawbacks of in vivo processes while harnessing the selectivity of enzymatic catalysis and iii) helps access an urgently needed drug candidate.

## Graphical abstract

The drug candidate and psychedelic mushroom product psilocybin was produced by multienzyme‐charged beads in a biocatalytic cell‐free approach. Using immobilized fungal indolethylamine biosynthetic and additional bacterial enzymes, these beads turned over 4‐hydroxy‐l‐tryptophan to psilocybin. This approach circumvents the drawbacks of in vivo processes while harnessing the selectivity of enzymatic catalysis

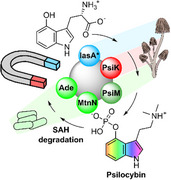

## 1 Introduction

**Figure 1:**
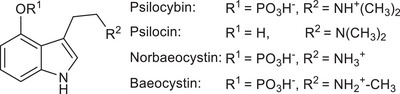
Chemical structures of *Psilocybe* tryptamine natural products.

Psilocybin (Figure 1) is the main natural product of *Psilocybe* mushrooms which cause powerful psychotropic effects when ingested.^[^ ^1^ ^]^ This 4‐*O*‐phosphorylated tryptamine is the precursor to the actual psychoactive compound, psilocin (Figure 1), which acts as a partial agonist on various serotonin (5‐hydroxytryptamine, 5‐HT) receptors, primarily the cortical 5‐HT_2A_ receptor.^[^ ^2^ ^]^ After having been banned for decades, psilocybin is seeing renewed interest as a candidate medication. Outcomes of Phase 2 clinical trials demonstrate high efficacy in the treatment of major depressive disorder and treatment‐resistant depression. The United States Food and Drug Administration therefore granted “Breakthrough Therapy” status to psilocybin.^[^ ^3^ ^]^ Other clinical trials focus on cancer‐related existential distress and substance dependencies as indications.^[^ ^4^ ^]^

The demand for psilocybin to support legitimate experimental and pharmaceutical purposes has been met by total chemical synthesis. Various protocols exist, including a recently published scalable cGMP‐compliant procedure.^[^ ^5^ ^]^ However, chemical synthesis may be limited by the complexity of the process and the generation of hazardous waste, making it less sustainable for large‐scale production. Furthermore, the chemical phosphorylation of psilocin to psilocybin in particular has been shown to be tedious, depending on several narrow critical process parameters to be achieved reproducibly at scale.^[^ ^5^ ^]^

The four enzymes catalyzing the biosynthesis in *Psilocybe* mushrooms have been identified, i.e., the l‐tryptophan decarboxylase PsiD, the P_450_ monooxygenase PsiH, the kinase PsiK, and the *N*‐methyltransferase PsiM.^[^ ^6^ ^]^ These findings set the stage for in vitro biocatalytic production of psilocybin. The procedures include the full enzymatic synthesis, using a step‐economic route beginning from 4‐hydroxy‐l‐tryptophan or from 4‐hydroxyindole and l‐serine.^[^ ^6^, ^7^ ^]^ as well as the PsiK‐mediated biocatalytic phosphorylation of synthetic psilocin.^[^ ^8^ ^]^ Alternatively, in vivo approaches with recombinant psilocybin‐producing microorganisms harboring the biosynthesis genes^[^ ^6^, ^9^ ^]^ were reported. The first heterologous production was shown in *Aspergillus nidulans*,^[^ ^10^ ^]^ later reproduced with yeast and *E. coli* as hosts and extended to make non‐natural analogs.^[^ ^11^ ^]^ Compared with cellular production in recombinant organisms, in vitro procedures offer i) endotoxin‐ and pyrogen‐free production conditions, which is preferred from a regulatory standpoint and for application in humans, as well as ii) high product specificity, as only a defined set of enzymes is present.

To perform a biocatalytic multi‐step synthesis, an efficient method is to use immobilized enzymes in a flow reactor.^[^ ^12^ ^]^ When immobilizing enzymes on solid support for this purpose, preserving the activity of the enzymes is a key consideration. One method for preserving the activity is to fuse the enzyme to a HaloTag protein, a small protein that covalently binds to a specific chloroalkane ligand, which can then be site‐selectively immobilized on a solid support bearing the cognate chloroalkane (available as beads or resin).^[^ ^13^ ^]^ As a proof of concept and to establish a foundation forcontinuously operated flow reactors, we report cell‐free psilocybin production using enzymes from various sources, covalently immobilized on a functionalized matrix.

## 2 Results and Discussion

### 2.1 Individual Activity Assays with HaloTag Fusion Proteins

**Scheme 1:**
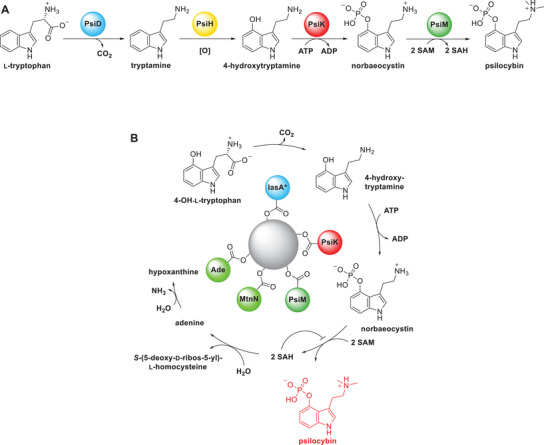
Cellular and biotechnological assembly of psilocybin. A) Biosynthetic sequence to psilocybin, intrinsic to *Psilocybe* mushrooms. B) Biocatalysis by co‐immobilized enzymes. PsiD, PsiK, PsiM designate *P. cubensis* l‐tryptophan decarboxylase, tryptamine‐4‐monooxygenase, 4‐hydroxytryptamine kinase, and norbaeocystin *N*‐methyltransferase, respectively. IasA*: *P. mexicana* aromatic amino acid decarboxylase/aromatic aldehyde synthase, engineered to replace residue Phe329 by tyrosine; MtnN and Ade: *Escherichia coli S*‐adenosyl‐l‐homocysteine nucleosidase and adenine deaminase, respectively, the grey sphere represents the HaloTag bead.

We aimed at reconstituting psilocybin biosynthesis (Scheme 1) from 4‐hydroxy‐l‐tryptophan using PsiD, PsiK and PsiM, thereby circumventing the step catalyzed by the notoriously insoluble PsiH monooxygenase and eliminating the need to add a cytochrome reductase to the system.^[^ ^6a^ ^]^ The enzymes were heterologously produced in *E. coli* KRX and fused to the HaloTag, analogous to similar previously reported applications of this technology.^[^ ^14^ ^]^ Unlike chelation with cations, this method covalently attaches enzymes to the matrix, preventing the leaching of heavy metals. In addition, the enzyme‐charged beads can be magnetically collected to facilitate product purification.

**Figure 2:**
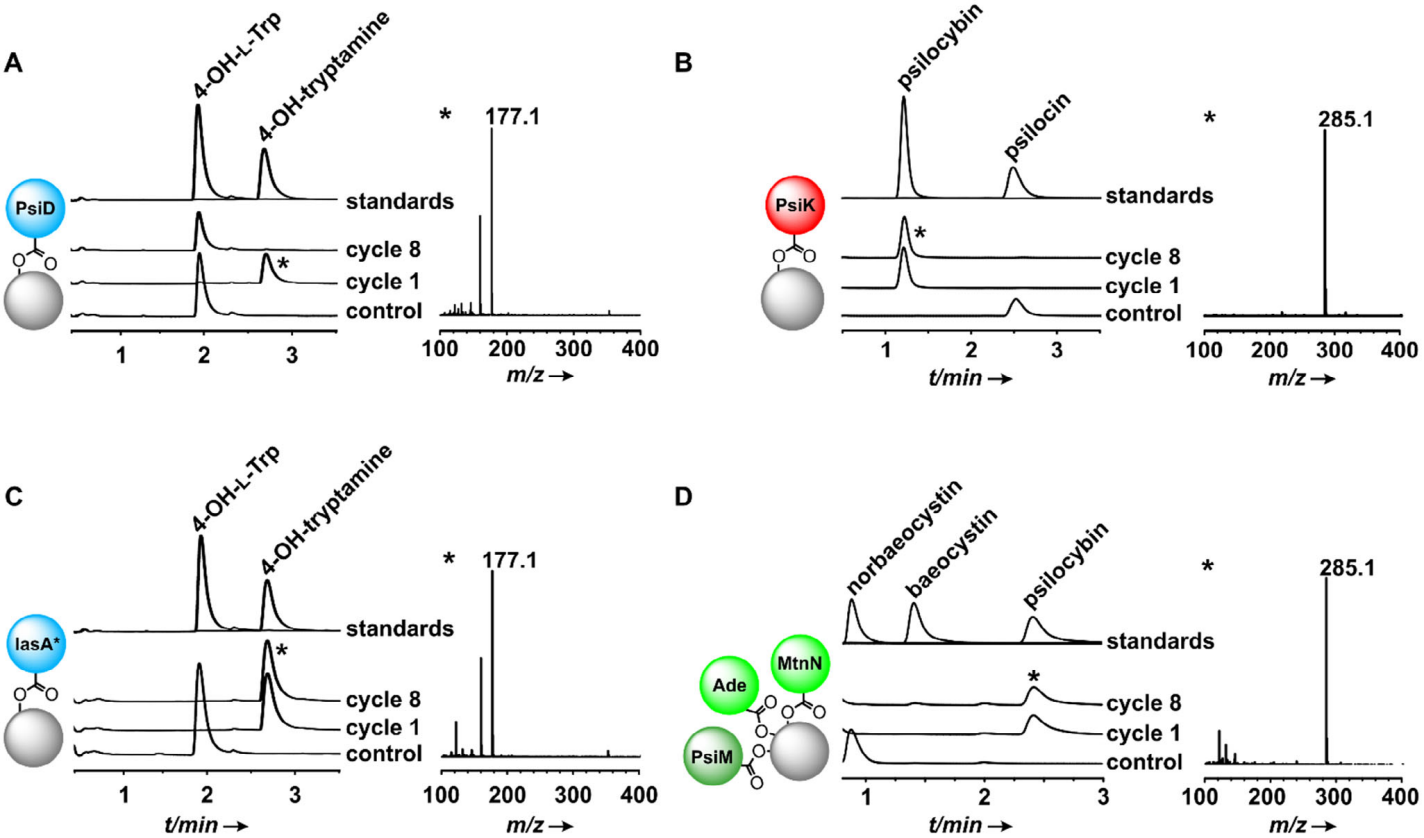
LC‐MS analysis of in vitro assays to determine the activity of individual halotagged immobilized biosynthesis enzymes (“cycle 1″) and their stability after eight consecutive reactions (“cycle 8″). Top traces show overlaid chromatograms of standards, bottom traces show negative controls with heat‐inactivated enzyme. Chromatograms were extracted from UV/Vis spectra at *λ* = 280 nm. Mass spectra were extracted from product peaks (indicated by asterisks) after the eighth cycle (except for PsiD). A): Decarboxylation of 4‐hydroxy‐l‐tryptophan by PsiD; B) psilocin phosphorylation by PsiK; C) decarboxylation of 4‐hydroxy‐l‐tryptophan by IasA* (replacing PsiD); D) N‐methylation of norbaeocystin to baeocystin and on to psilocybin by PsiM in the presence of *S*‐adenosyl‐l‐homocysteine nucleosidase (MtnN) and adenine deaminase (Ade).

In our work, the tagged and matrix‐bound enzymes were first tested individually to verify they had retained their catalytic activity. Both the pyridoxal‐5‐phosphate (PLP)‐independent gateway decarboxylase PsiD and the kinase PsiK showed (near‐)quantitative conversion (Figure 2) of the substrates added at a final concentration of 50 µm. To prevent PsiM inhibition by its second product *S*‐adenosyl‐l‐homocysteine (SAH), the methyltransferase assays were performed in the presence of N‐terminally HaloTagged *E. coli* SAH nucleosidase (MtnN) and adenine deaminase (Ade) that together irreversibly remove SAH from the reaction. Like with PsiD and PsiK, the HaloTag did not impact PsiM activity and sequential methylation of the primary amine norbaeocystin into the tertiary amine psilocybin was observed, while the intermediate secondary *N*‐methylamine baeocystin was only observed in trace amounts (Figure 2). As control, heat‐inactivated enzymes were used in parallel reactions. For additional control, the fusion enzymes were proteolytically cleaved by HaloTEV protease to elute them from the matrix. Subsequent SDS‐polyacrylamide gel electrophoresis indicated strong bands at the sizes expected for PsiK and PsiM, while PsiD recovery from the matrix resulted only in a faint band indicating non‐optimal covalent attachment to the matrix (Figure S1). This suggests that PsiD may not be as effectively immobilized as PsiK and PsiM, potentially affecting its stability and activity in the biocatalytic process.

### 2.2 Repeated use of Immobilized Enzymes

For the envisioned solid‐phase approach to produce psilocybin, the matrix‐bound enzymes PsiD, PsiK, and PsiM were tested individually for their ability to be reused. They were recovered from an assay, collected after the reaction, and reused, leading to eight consecutive reactions, each freshly set up. PsiM‐catalyzed reactions were again run in the presence of MtnN and Ade. After eight completed reaction cycles, both PsiK and PsiM catalysts still showed full activity (Figure 2).

Remarkably, solid phase‐bound PsiK turned out a highly reusable catalyst as it showed still quantitative turnover from psilocin to psilocybin even after as many as twenty reaction cycles (Figure S2). However, PsiD's activity had faded. Although the first cycle led to a 96% conversion, this value dropped to 26% after the fifth and < 2% after the eighth cycle. This finding suggests that it may have been compromised by immobilization in a way that PsiK and PsiM are not. PsiD is evolutionarily and mechanistically related to phosphatidylserine decarboxylases which autocatalytically remove their C‐terminal portion, the α‐subunit, which then serves as cofactor that binds the substrate.^[^ ^6^, ^15^ ^]^ As PsiD carries the tag at its N‐terminus, repeated recovery from the reactions may have insufficiently carried over the α‐subunit which may have caused the loss of its activity.

To address this apparent deficiency, we replaced PsiD by IasA from *P. mexicana*, an experimentally verified aromatic aldehyde synthase.^[^ ^16^ ^]^ These PLP‐dependent enzymes share a near‐indistinguishable evolutionary relationship with aromatic amino acid decarboxylases as a single amino acid residue determines the actual catalytic activity.^[^ ^17^ ^]^ We introduced a point mutation to replace this key residue, Phe329, by tyrosine. This engineered enzyme, hereafter referred to as IasA*, was produced in *E. coli* as a C‐terminal HaloTag fusion, immobilized on the Magne HaloTag Beads resin, and assayed for activity. It showed decarboxylase activity with 4‐OH‐l‐tryptophan (Figure 2). The immobilized version was also tested for repeated use and showed full activity after eight reactions (Figure 2).

### 2.3 Production of Psilocybin with Co‐Immobilized Enzymes

Continuing with the IasA*/PsiK/PsiM combination of biosynthetic enzymes, expanded by MtnN and Ade to remove the co‐product SAH, we evaluated the feasibility of an in vitro reaction to produce psilocybin, catalyzed by a single multi‐enzyme matrix all present in a single reaction. We assessed whether the loading of the beads with the tagged enzymes is reproducible. In a triplicate experiment, the covalently bound proteins from independent loading events were released by TEV protease and subjected to tryptic protein digestion. Subsequently, nano LC‐MS/MS was used to analyze the relative amounts of the released enzymes. The molar ratio of the enzymes PsiK, PsiM, IasA*, MtnN, and Ade was 37:20:11:13:19 (Figure S3). The highest batch‐to‐batch variability was ± 0.35%. In addition, an absolute photometric protein quantification was performed which indicated 19.6 (± 1.24) µg total protein per mg dried magnetic beads.

**Figure 3:**
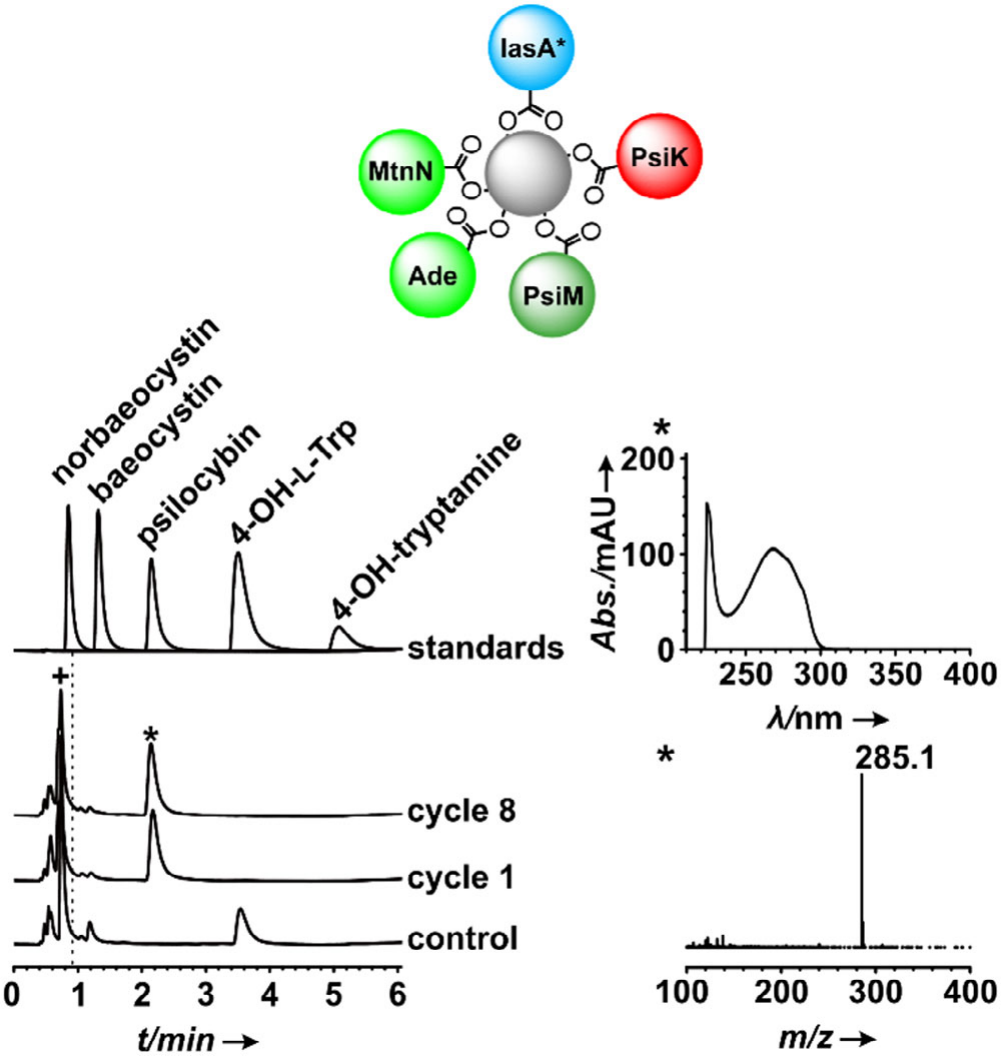
LC‐MS analysis of psilocybin production in vitro using immobilized enzymes. Chromatograms were extracted from UV/Vis spectra at *λ* = 280 nm. Upper trace: authentic standards of psilocybin, psilocin, 4‐hydroxy‐l‐tryptophan, and 4‐hydroxytryptamine (overlaid separate chromatograms); center trace: reaction with resin, charged with IasA*, PsiK, PsiM, MtnN, and Ade, and 4‐hydroxy‐l‐tryptophan as substrate; bottom trace: negative control with heat‐inactivated enzymes. + = injection peaks also containing excess ATP as substrate, hypoxanthine as final product, and other compounds. Right panels show the UV‐Vis and mass spectrum of the produced psilocybin.

The pathway was reconstituted beginning with 4‐hydroxy‐l‐tryptophan (50 µm) as substrate, which was readily accepted by IasA*, to eliminate the 4‐hydroxylation step catalyzed by the poorly soluble monooxygenase PsiH. Consequently, this approach makes a cytochrome P_450_ reductase as additional enzyme unnecessary. In the presence of the cofactors PLP, ATP, and SAM, psilocybin was observed as virtually exclusive indolethylamine product in Cycle 1 after 8 h (Figure 3), and constant yields across eight loading cycles (Figure S4). This finding underscores the cooperation between the participating *Psilocybe* and *E. coli* enzymes under homogeneous reaction conditions.

This study focused on the concerted use of immobilized biosynthesis enzymes to implement a fully biocatalytic, yet easy to handle process to synthesize psilocybin, a promising future medication against major depressive disorder. The multi‐enzyme in vitro biocatalysis represents a versatile method that avoids toxic waste and the use of heavy metals in chemical syntheses as well as potential and difficult‐to‐remove contaminants endotoxins or pyrogens carried over from a microbial host after an in vivo synthesis. Charging the beads is facilitated by highly selective covalent interaction to the target proteins directly in the crude lysate of the heterologous host.

Prior chromatography is not required which excludes carryover of Ni^2+^ or Co^2+^ from metal affinity chromatography columns while allowing for intensive wash steps that warrant a contaminant‐free reaction environment. Furthermore, the enzymes can be magnetically removed after the reaction which facilitates subsequent purification of the product. While the kinetics of PsiK, PsiM, MtnN, and Ade are known,^[^ ^8^, ^18^, ^19^ ^]^ future work needs to determine the values for IasA and IasA*. Our current results rely on a single species of charged beads, carrying multiple enzymes. However, the method can be varied by combining single‐enzyme beads which allows to compensate for kinetically slower, rate‐limiting steps.

Our method is not restricted to psilocybin, but offers the modularity and flexibility to easily swap or add enzymes. Future work will include TrpB, the substrate‐flexible *Psilocybe* tryptophan synthase to use l‐serine and substituted indoles as low‐cost starting material, e.g., to produce 4‐hydroxy‐l‐tryptophan in situ or to generate 6‐methylpsilocybin or isonorbaeocystin.^[^ ^7^, ^20^ ^]^ To supply SAM at a reasonable cost to our process, we also aim at integrating SAM production from ATP and l‐methionine by adding the *Psilocybe cubensis* SAM synthase SamS^[^ ^21^ ^]^ or *Escherichia coli* EcMAT.^[^ ^22^ ^]^ Similarly, the method is also applicable for only a single immobilized enzyme. Thus, tedious chemical syntheses are circumvented while integrating the simplicity of tryptamine chemistry,^[^ ^23^ ^],^ e.g., in case of 5‐methylpsilocybin and other synthetic non‐natural analogs.^[^ ^24^ ^]^ In particular, the extraordinary stability of PsiK that allowed for 20 consecutive reactions, lays the foundation for regioselective large scale 4‐*O*‐phosphorylation in continuously operated reactors.

The successfully completed proof of concept for biocatalytic psilocybin biosynthesis by solid phase‐supported enzymes lays the foundation for further in‐depth investigations of enzyme stability, consistency, increased enzyme loading, and purity across batches. These aspects warrant future investigations with a potential GMP‐compatible production and regulatory approval process in mind.

## 3 Conclusion

Psilocybin has (re‐)emerged as a promising candidate drug which translates into an increasing demand for drug substance to supply clinical trials. Besides total and chemoenzymatic syntheses in larger quantities ^[^ ^5^, ^8^ ^]^ our in vitro approach with immobilized biosynthetic enzymes adds to the cell‐free production options. Currently, campaigns are underway that aim at approval of psilocybin as a pharmaceutical in the United States and the European Union. We conclude that our results support these efforts by devising a scalable and universally applicable method for facile access to an urgently needed treatment against major depressive disorder.

## 4 Experimental Section

### Materials and general procedures

Chemicals, solvents, and media components were purchased from AstaTech, Roth, Sigma‐Aldrich, VWR. Authentic standards of baeocystin and norbaeocystin were synthesized as described.^[^ ^25^ ^]^ Plasmid isolation, DNA restriction, and ligation followed the instructions of the manufacturers of kits and enzymes (Macherey‐Nagel, Promega).

### Construction of plasmids

Plasmid pFN19K (Promega) served as vector for expression plasmids. The genes were inserted individually between the *Asi*SI and *Pme*I sites. To introduce these restriction sites, *psiD*, *psiK*, and *psiM* were amplified by PCR from plasmids pJF24, pJF23, and pFB13, respectively.^[^ ^6a^ ^]^ Plasmids pFB15 and pFB16 served to amplify genes for SAH nucleosidase (*mtnN*) and adenine deaminase (*ade*).^[^ ^20^, ^26^ ^]^ Ligation of the genes to pFN19K yielded expression plasmids pTS01 (*psiD*), pTS06 (*psiK*), pTS07 (*psiM*), pTS17 (*mtnN*), and pTS18 (*ade*) to produce N‐terminally halotagged proteins. To produce IasA* as a C‐terminally halotagged protein carrying a TEV recognition sequence at the fusion site, the *iasA* gene was PCR‐amplified from pPS66^[^ ^16^ ^]^ as two overlapping fragments to replace the phenylalanine by a tyrosine codon. The halotag gene was amplified from pFN19K and the four fragments were assembled by Gibson assembly, yielding plasmid pTS106. For PCR, 200 µm each dNTP, 10 ng template DNA, and 1 U *Pfu* DNA polymerase (Promega) in the buffer supplied with the enzyme, were used. Details on PCR conditions and oligonucleotides are provided in Tables S1 and S2. Following the PCRs, all products were purified by agarose gel electrophoresis and subsequent elution of the DNA with Promega's Wizard SV Gel and PCR Clean‐Up System. Expression plasmids pTS17 (*mtnN*) and pTS18 (*ade*) were created by joining the PCR product with pFN19K (co‐restricted by *Asi*SI and *Pme*I) using the NEBuilder kit (NEB), other expression plasmids were assembled by ligation (T4 DNA ligase, Promega) of *Asi*SI and *Pme*I‐generated sticky‐ends.

### Heterologous production and immobilization of PsiD, PsiM, PsiK, IasA*, MtnN, and Ade

To produce halotagged enzymes, *E. coli* KRX (Promega) was individually transformed with plasmids pTS01, pTS06, pTS07, pTS17, pTS18, and pTS106. The main cultures (1 L each) in LB medium, supplemented with 50 µg mL^−1^ kanamycin, were inoculated with a 5 mL overnight seed culture at 37°C and 180 rpm and induced with 0.1% l‐rhamnose after reaching an OD_600_ of 0.6. Subsequently, the temperature was lowered to 16°C, and the flasks were shaken for another 20 h before cell harvest by centrifugation. The biomass was resuspended in lysis buffer (7 mL per 1 L culture volume) containing 50 mm TRIS pH 8 and 150 mm NaCl. The cells were sonicated on ice, and the debris was removed by centrifugation for 20 min at 13,750 × g. To immobilize the fusion enzymes on the matrix (Magne HaloTag Beads, Promega), 20 µL of settled magnetic beads (from 100 µL slurry) were mixed with 800 µL cell lysate when a single enzyme was loaded (400 µL lysate per enzyme to simultaneously co‐charge the matrix with multiple enzymes) and shaken for 30 min. After discarding the flow through, the procedure was repeated for another 30 min. The immobilized enzymes were washed 10 times with 1 mL of the respective reaction buffer (Section 4.4) before substrate and cofactors (if applicable) were added. For control, aliquots of the loaded matrix were removed to release bound enzyme using the HaloTEV protease (Promega) for subsequent analysis by SDS‐polyacrylamide gel electrophoresis (Figure S1).

### In vitro assays with single immobilized enzymes

Triplicate reactions were set up in 50 mm phosphate buffer (pH 6.6 for PsiD and pH 7.0 for PsiK), or 50 mm TRIS‐HCl pH 8.0 (reactions with PsiM/MtnN/Ade or IasA*) to meet the pH optima of the individual enzymes. Indolethylamine substrates were added at 50 µm (PsiD: 4‐hydroxy‐l‐tryptophan, PsiK: psilocin, PsiM: norbaeocystin, IasA*: 4‐hydroxy‐l‐tryptophan). For PsiK assays, 100 µm ATP and 250 µm MgCl_2_ were used. For the methyltransferase assay, 300 µm SAM and *E. coli* MtnN and Ade, co‐immobilized with PsiM on the same beads, were added. IasA* assays were run in the presence of 5 µm PLP. Reactions were incubated with agitation for 30 min (PsiM for 8 h) at room temperature. Subsequently, the enzyme‐charged beads were trapped magnetically, before the supernatant was frozen in liquid nitrogen and lyophilized. Assay conditions for repeated enzyme use were as described above. Following an incubation cycle, the enzymes were trapped magnetically, the supernatant was removed and analyzed chromatographically, the enzyme‐charged beads were washed three times with reaction buffer prior to adding them to a new assay. Eight reactions were run with these enzymes, an additional set of PsiK assays included 20 consecutive reactions.

### In vitro assays with combined immobilized enzymes

The multi‐enzyme reactions (1 mL total volume) were run in triplicate and included IasA*, PsiK, PsiM, MtnN, and Ade. The proteins were produced individually, the lysates were combined and used to charge the Magne HaloTag Beads as described above. The reactions were set up in 50 mm TRIS‐HCl buffer (pH 8) and 100 µm ATP, 250 µm MgCl_2_, 300 µm SAM, 5 µm PLP, and 50 µm 4‐hydroxy‐l‐tryptophan. Settled beads (20 µL), charged with the respective enzymes, were added to the reactions. They proceeded for 8 h, shaken at 200 rpm, at room temperature. Subsequently, the enzyme‐charged beads were trapped magnetically and the liquid was frozen in liquid nitrogen, lyophilized, and subsequently dissolved in 100 µL methanol, centrifuged, and used for chromatography (below). After washing the beads three times with buffer, new buffer, substrate, cosubstrate, and cofactors were added to the beads as described above for single‐enzyme assays. Heat‐inactivated bead‐bound enzymes were negative controls.

### Protein quantification and analysis

The immobilized proteins were removed at room temperature for 2 h with 5 U µL^−1^ HaloTEV protease (Promega) in 500 µL 100 mm ammonia bicarbonate buffer, shaking the reaction at 200 rpm. For an in‐solution digest, proteins were solubilized in 100 µL 100 mm ammonium bicarbonate and mixed with 100 µL 2,2,2‐trifluoroethanol. Cysteine thiols were reduced and carbamidomethylated in one step for 30 min at 70°C by addition of each 4 µL of 500 mm tris(2‐carboxyethyl)phosphine and 625 mm 2‐chloroacetamide per 200 µL sample. Subsequently, sample solutions were evaporated to approximately 100 µL. Proteins were precipitated with methanol (MeOH)/chloroform/H_2_O, following a published procedure^[^ ^27^ ^]^ and resolubilized in 100 mm tetraethylammonium bicarbonate in 5:95 trifluoroethanol/H_2_O (v/v). Proteins were digested for 18 h at 37°C after addition of Trypsin/Lys‐C mix at a protease‐to‐protein ratio of 1:25. Tryptic peptides were dried in a vacuum concentrator and resolubilized in 30 µL of 0.05% trifluoroacetic acid in H_2_O:acetonitrile (ACN) 98:2 (v/v), filtered through membrane spin filters (10 kDa cut off). The filtrate was transferred to HPLC vials and injected into the chromatograph (below). Each sample was measured in triplicate (three analytical replicates). Photometric protein quantification was performed using the Pierce BCA Protein Assay Kit (Thermo).

### Liquid chromatography and mass spectrometry

To analyze products of single‐enzyme assays with halotagged PsiD or IasA*, an Agilent 1290 Infinity II UHPLC‐MS instrument was used, equipped with a diode array detector (DAD) and interfaced to a 6130 quadrupole mass detector, run in positive mode and using an ESI source. The chromatograph was fitted with a Supelco Ascentis Express F5 column (100 × 2.1 mm, 2.7 µm particle size) and a guard column. Separation was at 50°C (flow: 0.5 mL min^−1^). Solvent A was 0.1% aqueous formic acid (FA), solvent B was MeOH. A linear gradient was applied (% B): initially 10%, within 8 min to 100%. For PsiK assays, the same chromatograph was fitted with a Phenomenex Luna Omega Polar C18 column (50 × 2.1 mm, 1.6 µm particle size) and an appropriate guard column. Separation was at 25°C and a flow of 0.5 mL min^−1^. Solvent A was 0.1% aqueous FA, solvent B was ACN. A linear gradient was applied (%B): initially 1%, within 3 min to 5%, and within further 1 min to 100%. To analyze PsiM/MtnN/Ade assays and psilocybin producing multi‐enzyme assays, a Supelco Ascentis Express F5 column was used (100 × 2.1 mm, 2.7 µm particle size), thermostatted at 35°C. Solvent A was 0.1% aqueous FA, solvent B was MeOH. A linear gradient at a flow of 0.6 mL min^−1^ was applied (%B): 0–4 min 2%, within further 10 min to 100%.

Proteins were analyzed on a Thermo Fisher Scientific Ultimate 3000 nano RSLC chromatograph connected to a Q‐Exactive HF mass spectrometer. Peptide trapping for 5 min on an Acclaim Pep Map 100 column (2 cm × 75 µm, 3 µm) at 5 µL min^−1^ was followed by separation on an µPAC neo 110 column. Eluent A was 0.1% aqueous FA, eluent B was 0.1% (v/v) FA in 90:10 ACN:H_2_O. Gradient elution was performed using the following gradient: 0 min at 4% B and 750 nL min^−1^, 10 min at 6.5% B and 750 nL min^−1^, 12 min at 7% B and 300 nL min^−1^, 85 min at 25% B and 300 nL min^−1^, 130 min at 50% B and 300 nL min^−1^, 135 min at 96% B and 300 nL min^−1^, 138–140 min at 96% B and 750 nL min^−1^, 140.1–150 min at 4% B and 750 nL min^−1^. Positively charged ions were generated at spray voltage of 2.2 kV using a stainless steel emitter attached to the Nanospray Flex Ion Source. The quadrupole/orbitrap instrument was operated in data‐dependent acquisition mode. The top 15 most abundant precursor ions per scan cycle underwent HCD fragmentation. Precursor ions were monitored at *m*/*z* 300–1500 at a resolution of 120,000 FWHM (full width at half maximum) using a maximum injection time (IT_max_) of 120 ms and an automatic gain control target of 3 × 10^6^. Precursor ions with a charge state of *z* = 2–5 were filtered at an isolation width of *m*/*z* 4.0 amu for further fragmentation at 30% HCD collision energy. MS^2^ ions were scanned at 15,000 FWHM (IT_max_ = 80 ms, AGC = 2 × 10^5^). Dynamic exclusion was 30 s.

### Protein database search

Tandem mass spectra were searched against the UniProt databases (retrieved on August 19, 2024) of *E. coli* K12, supplemented by the protein sequences of halotagged Ade, IasA*, MtnN, PsiK, and PsiM, using Proteome Discoverer (PD) 3.1 (Thermo) and the database search algorithms (threshold search engine scores in parenthesis) Mascot 2.8 (>30), Comet (>3), MS Amanda 3.0 (>300), Chimerys (>2), Sequest HT (>3) with and without INFERYS Rescoring with Precursor Detector node. Two missed cleavages were allowed for the tryptic digestion. The precursor mass tolerance was set to 10 ppm and the fragment mass tolerance to 0.02 Da. Modifications were defined as dynamic methionine oxidation, phosphorylation of serine, threonine, and tyrosine, protein N‐terminal acetylation with and without loss of methionine as well as static cysteine carbamidomethylation. A strict false discovery rate < 1% (peptide and protein level) was required for positive protein hits. The Percolator node of PD 3.1 and a reverse decoy database was used for *q*‐value validation of spectral matches. Only Rank 1 proteins and peptides of the top scored proteins were counted. Label‐free protein quantification was based on the Minora algorithm of PD 3.1 using the precursor abundance based on intensity and a signal‐to‐noise ratio > 5. The relative percentage distribution of Ade, MtnN, lasA*, PsiM, and PsiK was calculated based on the abundance counts (spectral counting) per protein divided by the respective length of the protein sequence (number of amino acids). The sum of these relative spectral counts of the five proteins was defined as 100%, and the percentage was determined for the individual values. The mass spectrometry proteomics data have been deposited to the ProteomeXchange Consortium via the PRIDE ^[^ ^1^ ^]^ partner repository with the dataset identifier PXD061303.

## Conflict of Interests

The authors declare no conflicts of interest.

## Supporting information



Supporting information
