# Supplementary material for: In Vitro Psilocybin Synthesis by Co‐Immobilized Enzymes
Source: Chemistry. 2025 Apr 21;31(29):e202501037. doi: 10.1002/chem.202501037 (PMC12099186; doi:10.1002/chem.202501037)
Supplement: Supplementary file 1 — Supporting information [file CHEM-31-e202501037-s001.pdf]

Table of Contents

Figure S1. SDS-polyacrylamide gel electrophoresis of eluted enzymes 2

Figure S2. Chromatograms of PsiK reactions after extended use 3

Figure S3. Quantification of immobilized enzymes 4

Figure S4. Quantification of psilocybin production *in vitro* across substrate loading cycles 5

Table S1. PCR conditions........ 6

Table S2. Oligonucleotide primers........ 7

References. 8

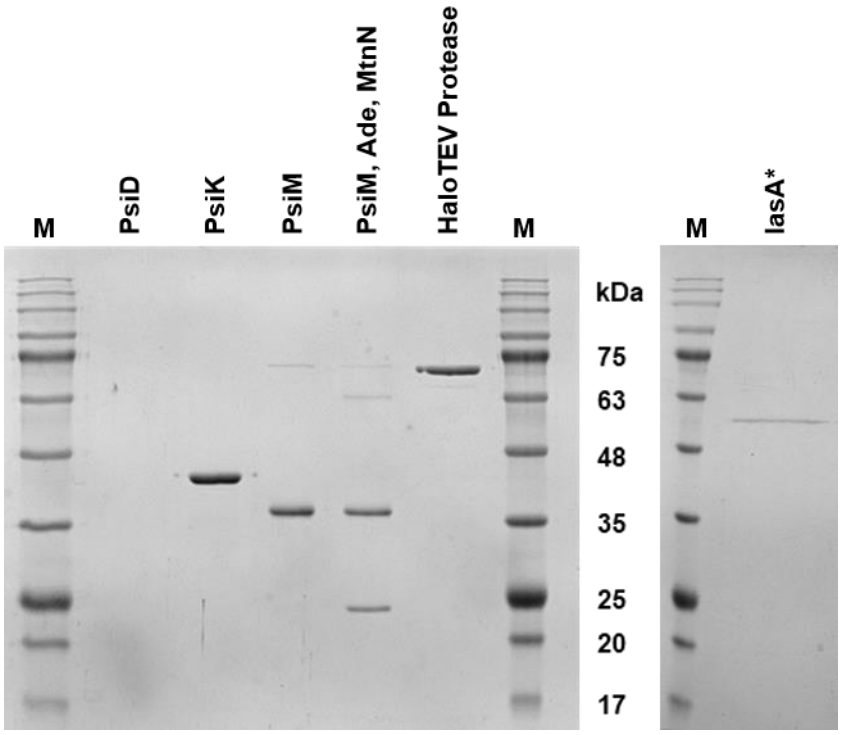

Figure S1. SDS polyacrylamide gel electrophoresis (12%) of enzymes eluted from the solid-phase matrix by HaloTEV protease cleavage. Calculated masses (in kDa) of the eluted proteins are 50.2 (PsiD); 41.0 (PsiK); 35.0 (PsiM); 25.0 (Ade); 64.4 (MtnN); and 56.6 (IasA*) M: Molecular weight marker, masses are indicated.

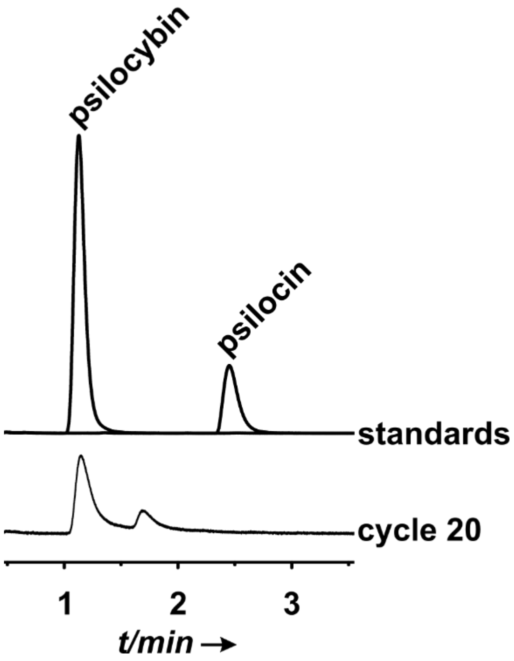

Figure S2. LC-MS analysis of quantitative psilocin phosphorylation into psilocybin, catalyzed by PsiK in the 20th consecutive reaction cycle (i.e., the immobilized enzyme had been used in and recovered from reactions 19 times before). The chromatograms were extracted at λ = 280 nm from diode array data. The peak at t_R = 1.7 min (*m*/*z* 137.1 [*M*+H]$^+$) is 1,4,5-oxadithiepane that formed due to the presence of β-mercaptoethanol in the PsiK reactions.[1]

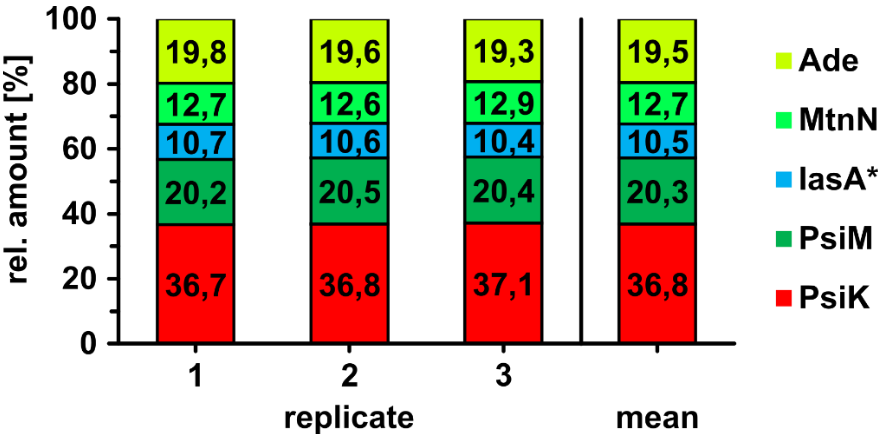

Figure S3. Quantification of enzymes immobilized on magnetic beads. The covalently bound enzymes from three independent bead loading events were released by TEV protease and analyzed. Shown is the molar ratio of the released enzymes. Enzyme names: Ade: adenine deaminase; MtnN: *S*-adenosyl-L-homocysteine nucleosidase; IasA*: L-tryptophan decarboxylase; PsiM: *N*-methyltransferase; PsiK: 4-hydroxytryptamine kinase.

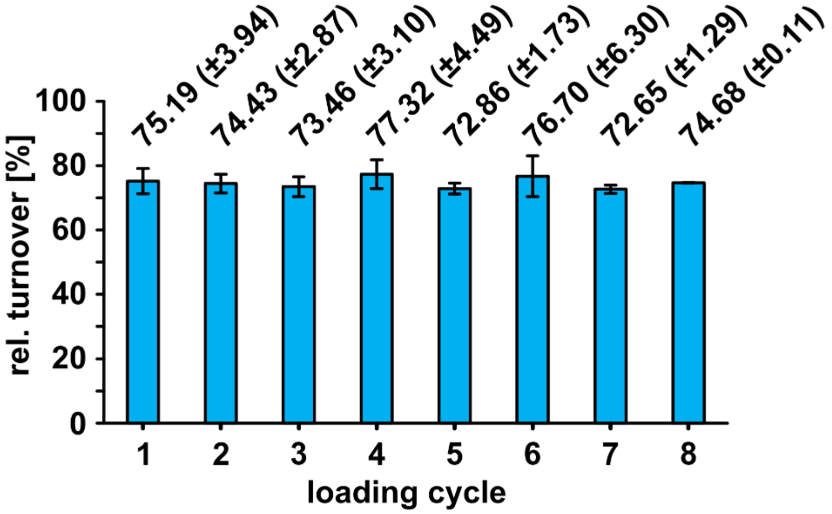

Figure S4. Quantification of psilocybin production *in vitro* across substrate loading cycles, (re-)using the multi-enzyme magnetic beads. Relative values are referenced to a theoretical quantitative substrate turnover, assuming spontaneous chemical degradation of intermediates and substrates did not occur. Psilocybin concentrations were determined using a calibration curve. Coupled assays were conducted in triplicate, error bars depict standard derivation.

Table S1. PCR parameters. All reactions included a final elongation (5 min at 72 °C). Oligonucleotide sequences are listed in Table S2.

Amplified gene	Template	Thermal cycling	Oligonucleotides	Expression plasmid
psiD	pJF24[1]	Initial hold: 98 °C for 30 s<br>30 cycles of 98 °C for 10 s, 68 °C for 20 s, 72 °C for 120 s	oTS04/oTS05	pTS01
psiK	pJF23[1]	Initial hold: 98 °C for 30 s<br>30 cycles of 98 °C for 10 s, 60 °C for 15 s, 72 °C for 110 s	oTS45/oTS46	pTS06
psiM	pFB13[1]	Initial hold: 98 °C for 30 s<br>30 cycles of 98 °C for 10 s, 60 °C for 15 s, 72 °C for 110 s	oTS47/oTS48	pTS07
*iasA**	pPS66[2]	Initial hold: 95 °C for 60 s<br>30 cycles of 95 °C for 30 s, 60 °C for 30 s, 72 °C for 150 s	oTS616/oTS583 (5’-terminal portion) and oTS582/oTS617 (3’-terminal portion)	pTS106
mtnN	pFB15[3]	Initial hold: 98 °C for 30 s<br>30 cycles of 98 °C for 10 s, 60 °C for 15 s, 72 °C for 110 s	oTS178/oTS179	pTS17
ade	pFB16[3]	Initial hold: 98 °C for 30 s<br>30 cycles of 98 °C for 10 s, 60 °C for 15 s, 72 °C for 110 s	oTS180/oTS181	pTS18
*halotag**	pFN19K*	Initial hold: 95 °C for 60 s<br>30 cycles of 95 °C for 30 s, 60 °C for 30 s, 72 °C for 120 s	oTS219/oTS144	-
pFN19K*	pFN19K*	Initial hold: 95 °C for 60 s<br>30 cycles of 95 °C for 30 s, 60 °C for 30 s, 72 °C for 6:20 min	oTS141/oTS220	-

* Commercial source (Promega)

Table S2. Oligonucleotides used for PCR. The restriction sites of *Pme*I (GTTTAAAC) and *Asi*SI (GCGATCGC) are underlined, the nucleotides in bold in oTS582 and oTS583 represent the exchanged codon in *iasA*. Double underlined sequence portions represent the overlaps for Gibson assembly in oligonucleotide pairs oTS141 and oTS144, oTS219 and oTS617, as well as oTS220 and oTS616. Oligonucleotides oTS582 and oTS583 overlap across their full length.

Oligonucleotide	Sequence (5' to 3')	Amplified gene
oTS04	TATATAGCGATCGCCATGCAGGTGATACCC	*psiD*
oTS05	TATATAGTTTAAACCTAAGCCTTTAGAGCAGC	*psiD*
oTS45	TATATAGCGATCGCCATGGCGTTCGATCTCAAG	*psiK*
oTS46	TATATAGTTTAAACTTACGCAGTGGATGATTCCTTC	*psiK*
oTS47	TATATAGCGATCGCCATGCATATCAGAAATCCTTACC	*psiM*
oTS48	TATATAGTTTAAACCTAGAAAAGAGAGCTGAGCTCG	*psiM*
oTS141	TAGGTTTAAACGAATTCGGGCTCG	pFN19K backbone for pTS106
oTS144	CCCGAATTCGTTTAAACCTAGCCGGAAATCTCGAGCG	halotag for pTS106
oTS178	AGAGCGATAACGCGATCGCCATGAAAATCGGCATCATTGGTGC	*mtnM*
oTS179	GAGCCCGAATTCGTTTAAACTTAGCCATGTGCAAGTTTCTGC	*mtnN*
oTS180	AGAGCGATAACGCGATCGCCATGAATAATTCTATTAACC	*ade*
oTS181	GAGCCCGAATTCGTTTAAACTTATTCCGTGACTTCC	*ade*
oTS219	GAGGATCTGTACTTTCAGAGCATGGCAGAAATCGGTACTGGCTTTCCATTCG	halotag for pTS106
oTS220	CTAAATTGTTCGATATCCATGATGAATTCTCCTTATTCTATAGTGTCACC	pFN19K backbone for pTS106
oTS582	GGATATTACACCTCCG**TAC**CTCAGAAC	*iasA*
oTS583	GTTCTGAG**GTA**CGGAGGTGTAATATCC	*iasA*
oTS616	TAGAATAAGGAGAATTCATCATGGATATCGAACAATTTAGAAAAGC	*iasA*
oTS617	GCTCTGAAAGTACAGATCCTCACCAACTATTGCCATTCCG	*iasA*

References

[1] J. Fricke, F. Blei, D. Hoffmeister, *Angew. Chem. Intl. Ed*. **2017**, *56*, 12352-12355; *Angew. Chem*. **2017**, *129*, 12524-12527.
[2] P. S. Seibold, S. Dörner, J. Fricke, T. Schäfer, C. Beemelmanns, D. Hoffmeister, *Fungal Biol. Biotechnol.* **2024**, *11*, 4.
[3] J. Fricke, A. Sherwood, R. Kargbo, A. Orry, F. Blei, A. Naschberger, B. Rupp, D. Hoffmeister, *ChemBioChem* **2019**, *20*, 2824-2829.
